# Using a Model Created Using a 3D Printer to Mould a Grey Cast Iron Casting

**DOI:** 10.3390/ma17164033

**Published:** 2024-08-14

**Authors:** Karel Ráž, Zdeněk Chval, Jiří Kořínek

**Affiliations:** Faculty of Mechanical Engineering, Regional Technological Institute, University of West Bohemia, Univerzitni 2732/8, 301 00 Plzen, Czech Republic; zdchval@fst.zcu.cz (Z.C.); jkorinek@fst.zcu.cz (J.K.)

**Keywords:** 3D print, cast iron, forming, turbine

## Abstract

This article deals with the possibility of using 3D-printed models as an input for the production of a mould for cast iron castings. This new progressive process is significantly faster (with sufficient accuracy) compared to the current way of making models for moulds. The need to create a wooden model is removed by this process. The quality of this wooden model was highly dependent on the experience and qualifications of the worker. This article describes the manufacturing process of the model and mould in detail. The key dimensions of the final parts are compared with the model and, thus, the accuracy of the chosen procedure is verified. A 3D-printing technology known as Multi Jet Fusion (MJF) was used to produce the model. The material used for the production of the model is polyamide PA12 with 40% glass fibre filling. This material has sufficient structural and strength properties to be used for the given application. Taking into account the dimensions of the part and the printing space of the printer, it was necessary to structurally modify and divide the part. The inlet cone of a turbine is used as an example This cone is produced from grey cast iron as standard.

## 1. Introduction

Due to current technological and design possibilities, the number of products produced using the technology of classical casting from cast irons has definitely been greatly reduced [1,2]. This has been caused, for example, by more accessible technology for cutting sheet-metal parts using a laser or plasma [3,4]. Sometimes it is possible to replace parts of the products with a relatively precise welded structure. Smaller companies focussed on metal piece production almost never use any other technology [5].

One of the reasons for this, in addition to the above-mentioned simplification, is the high technological demand, time consumption and financial demand for the production of classic castings [6,7]. This aspect is also reflected in the smaller number of foundries at the present time. Foundries deal mostly with the piece production of castings weighing up to approximately 500 kg [8]. Technologically outdated operations have not stopped the current pressure of competition, mainly from the point of view of the above-mentioned new technologies [9].

Another aspect is that society is moving in a direction rather distant from crafts, which means that these operations suffer from a lack of qualified or long-term, experienced workers [10,11].

One of the important aspects in the production of a casting is the creation of a model used to create, for example, a sand mould [12,13]. A classic, well-established procedure is the creation of a model from wood, by so-called wood-modellers using procedures like machine processing or, for more complex-shaped parts, using manual work, wherein the skill of the given wood modeller has to be reflected [14].

An important aspect is also the experience of the modeller in relation not only to the creation of the mould, but also to the actual casting and casting technology [15,16]. There is interesting technology which is suitable for direct 3D printing for underwater applications, which can be used, but there is limitation due to high abrasive loading of these parts [17]. Compared to this, the direct-printing method is the procedure described by the authors for those still considering casting production (due to a higher number of material types).

Currently, it is possible to create a model using 3D printing by using one of the available printing technologies. This modern approach to model preparation highly reduces the delivery time. On the other hand, it is necessary to have a suitable 3D printing technology. It has to be mentioned that there are an increasing number of factories focussed on 3D printing technology, according to a 3D model given by a customer. A case study performing a comparison between the regular wooden model production and 3D-printed polymer model production was undertaken. The main parameters for comparison were production time [hours] and accuracy with respect to the initial dimensions [deviations in mm]. Data for the wooden model were given by the qualified company producing the wooden models. The usage of a 3D-printed model can decrease the delivery time required for the model by up to 50%.

## 2. Entry Requirements—Specified Geometry and Usage

**Figure 1:**
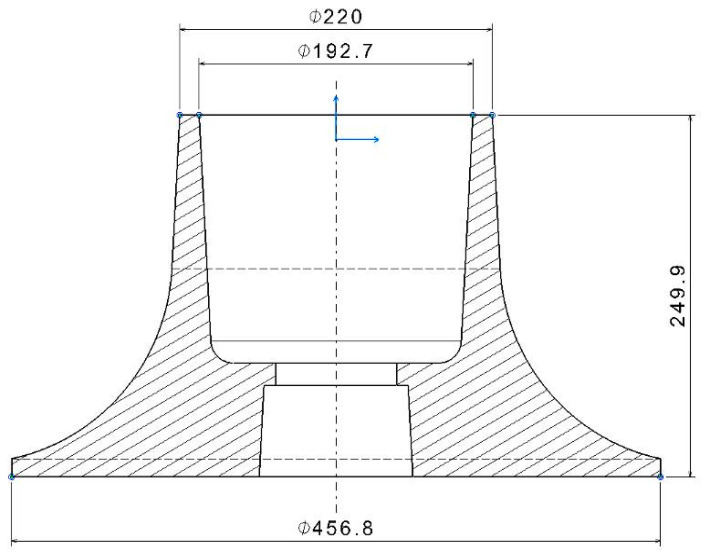
Dimension of the part.

Due to current technological and design possibilities, the area of use of products made using the technology of casting from cast irons has definitely been greatly reduced [18,19]. There are some parts, like one shown in the Figure 1, which are not possible to produce by any other technology [20,21]. Only casting is suitable [22]. The rotational shape, with a diameter up to 457 mm and height of 250 mm, has suitable dimensions for casting technology. The part shown as a benchmarking example is the cone used for a water-turbine inlet [23,24].

This part serves only as a shaped segment to direct water flow to the impeller [25]. Therefore, no significant strength requirements are placed on it [26,27]. On the other hand, due to the use of the mentioned turbine, with a total a slope of more than 10 m, it is possible that there may be signs of frictional cavitation, or abrasion of surface from polluted water—for example, drifting sand—in the long-term horizon of use [28,29]. 

**Figure 2:**
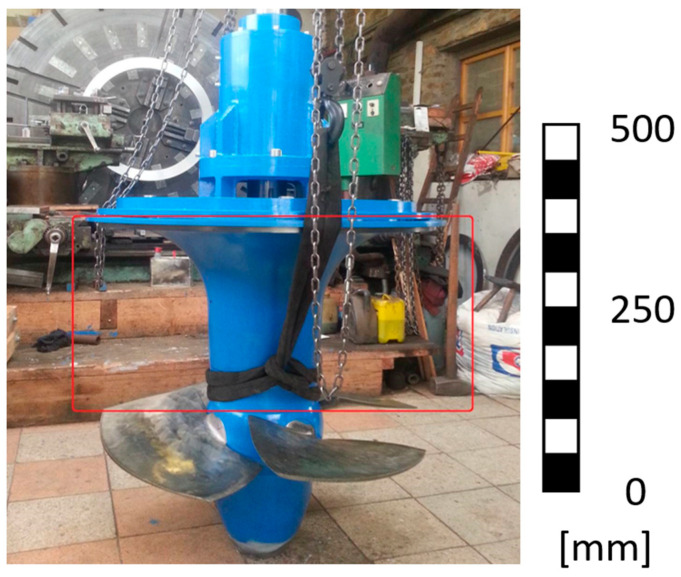
Picture of assembled Kaplan turbine with inlet cone marked in red.

The picture below (Figure 2) shows the Kaplan turbine assembly with the inlet cone marked in red. The shown turbine assembly has an 860 mm diameter impeller.

A Kaplan turbine is a type of reaction turbine used for low-head hydroelectric installations, known for its high efficiency across a wide range of flows. The application is considering the maximal power output 34 kW, with flow 2.3 m^3^/s. The efficiency of this turbine is around 85%. 

## 3. Processing in a 3D Environment—3D Model

**Figure 3:**
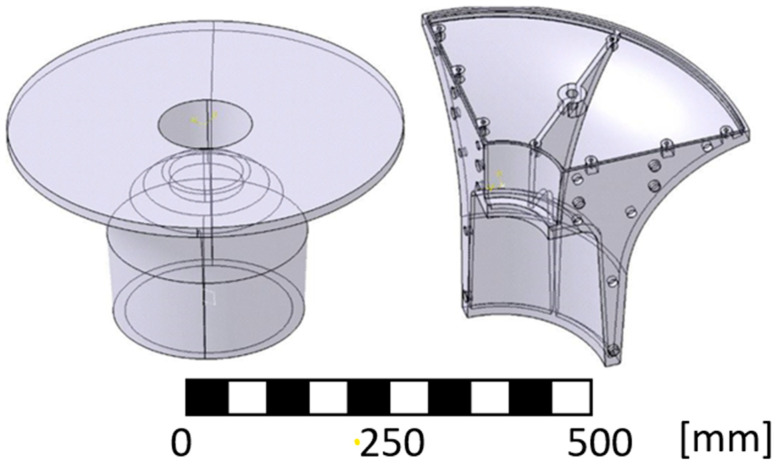
3D model in CAD software Catia v5-3DX.

The 3D model was created based on the specified geometry of the final machined product. To this geometry (Figure 3), 5 mm was added all over the outer shell for machining and then this model was enlarged by an assumed shrinkage of 3%, i.e., to a size of 103% [30,31]. This value turned out to be too large during implementation. The real value of the shrinkage of the casting due to cooling was around 1%.

However, the work on the 3D model itself did not end there. It was necessary to adapt this volume model for the possibilities of printing on a 3D printer.

The *.step file was used to transfer the data to the 3D printer, in order to ensure maximal accuracy. The transfer to the facet body was performed during the 3D printer preprocessing and the number of facets was around 1.5 million. 

## 4. Printing the Model on a 3D Printer and Its Completion and Modifications

The Multi Jet Fusion (MJF) 3D printing method used in this research relies on the sintering of powder material. This technology does not fit into one of the printing methods defined by the International Standards Organization (ISO), but it can be considered as part of binder jetting. Various materials can be used, but for this research, PA12GB (a polyamide with 40% glass beads) was selected [32,33]. This material has sufficient strength properties for this application [34]. The tensile strength of this material is 30 MPa, melting temperature is 186 °C, tensile modulus is 2800 MPa horizontal and 2900 MPa in a vertical direction and elongation at break is 6.5°. This technology was used because of maximal isotropic behaviour compared to other printing methods. It also has quite a short delivery time for final parts.

Unlike laser powder sintering, the 3D printer utilizes HP’s Multi Jet Fusion technology. This technology employs a fusion agent to define the shape of the resulting part, aiding in the absorption of infrared radiation into the plastic powder. A fine layer of powder is spread over the entire printing surface and heated to a temperature near its melting point. As the print head moves across the surface, it applies droplets of the fusing agent in the desired shape for each layer. An infrared lamp then heats the printing layer, causing a local increase in temperature above the material’s melting point. The printing surface is lowered by one layer, and the process is repeated [35,36,37].

**Figure 4:**
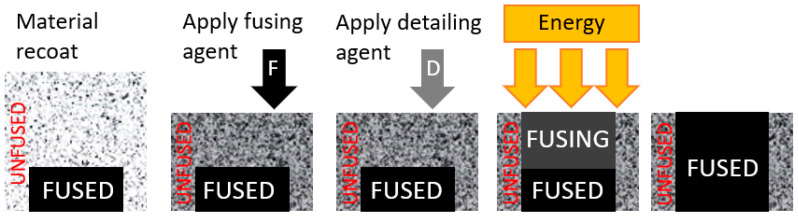
Principle of the MJF technology.

Following Figure 4 illustrates this process in detail. First, the material is deposited on the work surface. Then, the Fusion Agent (F) is selectively applied where the powder particles need to be joined. Next, the Detailing Agent (D) is selectively applied to areas where the fixing effect should be reduced or enhanced. In this example, the detailing agent will limit melting to the boundaries, creating parts with sharp, smooth edges. The work surface is then exposed to melting energy, resulting in a layer of bonded material where the fusion agent was applied. This process repeats until the entire part is completed.

The whole printing process takes around 16 h, with 32 h of cooling and postprocessing. This time is much lower compared to the delivery time of the wooden models (in days or weeks). There is a big advantage, which is that the operator does not need to manually operate the 3D printer for the entire time (the printer is working independently), compared to the wooden model operator, who has to spend 100% of this time with the part. The final surface quality was around Ra 3.2, which is sufficient with respect to the machining following the casting. There is a possibility (not necessary in this application) of using the chemical vapourization as a smoothing method. This smoothing can achieve Ra 1.6 (depending on the size and shape). Due to the use of the MJF technology, there was no need to use additional structural supports.

This technology has a positive environmental impact because it reduces material waste and it also allows production of more complex geometries with a higher level of customization. There are some 3D printing technologies which are able to use plastic waste as an input for material used within these technologies. Considering the large amount of plastic waste, this is a great advantage.

**Figure 5:**
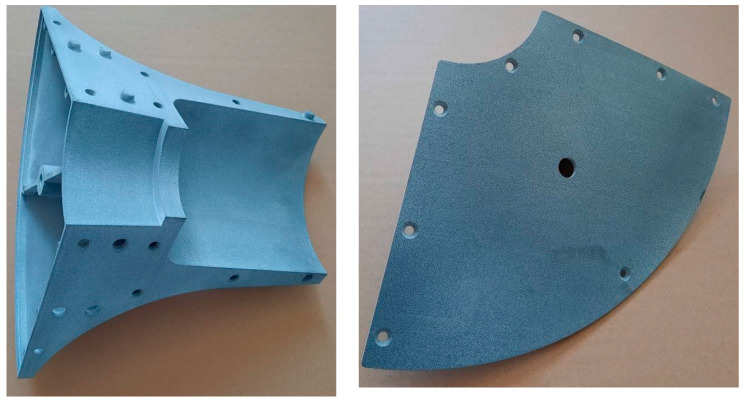
Quarter of printed model segment and segment cap.

**Figure 6:**
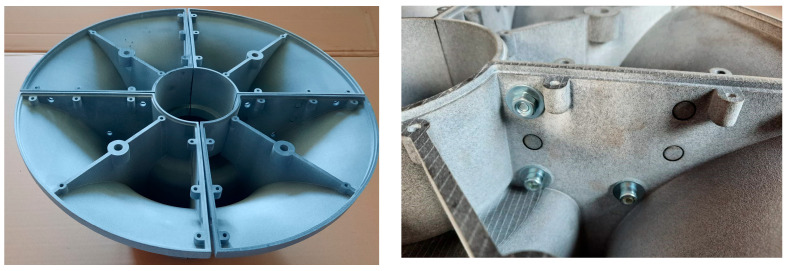
Assembled model segments and detail of the connection of the individual segments.

As it is written above, the 3D model was created based on the specified geometry. The model had to be divided into several parts due to the overall size. Quartering of the geometry was undertaken. The contact surfaces were pierced with spikes for splicing with the adjacent part. In addition, this surface was also drilled with holes for connection using five M6 screws so that each surface could be connected. Another modification of the model was the creation of a shell with a wall thickness of 3 mm (see Figure 5 and Figure 6) [38]. 

This resulted in material savings. At the same time, it was necessary to make the above model open in order to remove unnecessary printing material. Due to this fact, it was necessary to create a shaped connection for fitting the lid. Furthermore, the empty space needed to be ribbed—here using one rib, which later turned out to be a weak point in the design [39]. In addition, it was necessary to place a “spout” in the rib for the threaded hole necessary to remove the model from the mould. After practical experience, it would be advisable to consider the placement of a metal “clamp” with a thread [40,41,42].

**Figure 7:**
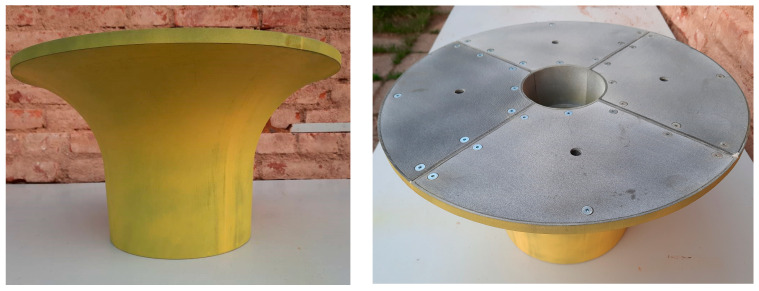
Finished model after grinding and cleaning.

After assembling all the parts into one whole, the model was connected at the joints using a two-component polyurethane sealant and sanded (Figure 7). The model was then painted with a two-component polyurethane paint to smooth the surface, and then the entire surface was lightly sanded with an emery cloth. The model itself was sandblasted after the printing was completed to remove excess remains of the printing material, and this sandblasting made the surface relatively rough. The above treatment with two-component paint softened the surface, which is desirable during the process of removing the model from the mould.

## 5. Final Product from Cast Iron

**Figure 8:**
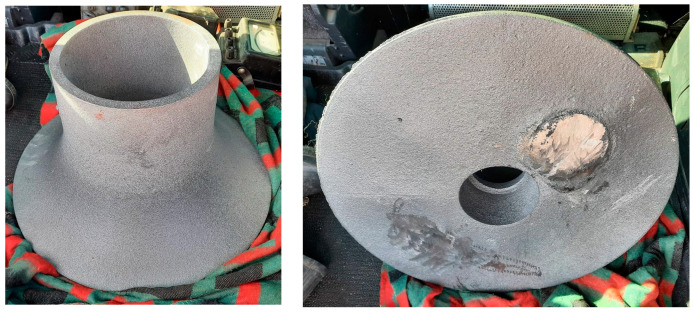
Final product from cast iron.

The casting itself was ready in about two weeks after the production order. The weight of the casting was 54 kg. In the foundry, the casting was removed from the sprue system and sandblasted. The casting itself showed no surface defects (Figure 8).

According to information from the foundry, everything was carried out according to established procedures and assumptions, and the model therefore fully matched the traditional wooden model in terms of useful properties. It should be noted that at the beginning of the process, i.e., entering into production, it was necessary to overcome a certain historical dogma, and the assumption that the model must either be wooden or it would not work.

In the photo shown above, the only significant error in the creation of the model is obvious. Due to the construction of the model, i.e., an empty volume with ribs and lids, when the moulding mixture was rammed, the lids were deformed (bent) in areas where they were not supported by said ribs. The largest deformation reached a value of approximately 2.5 mm from the flat surface. Since the model had a total allowance of 5 mm on each surface, this deformation of the model is not fatal and will not affect the final product.

## 6. Verification of the Geometric Parameters of the Final Product in Contrast with the 3D Model and the Print

Undoubtedly, interesting parameters in the production of cast iron are the comparison of the desired geometric values with the actual values achieved.

**Figure 9:**
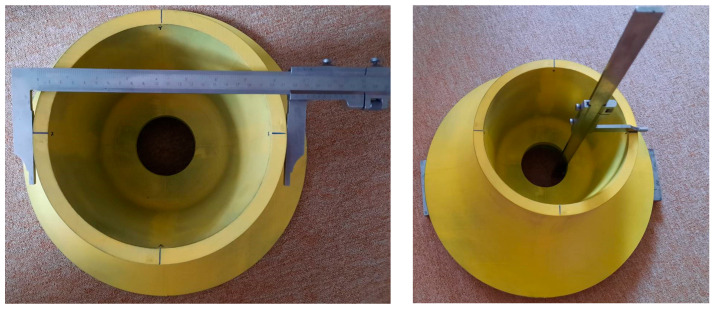
Example of analysed dimensions.

For a simple comparison of the results with the requirements, four basic dimensions were measured, an example of which is shown in the following photos. The measurement results (with measurement method in Figure 9) are entered in the Table 1, shown below.

From the table shown above, it is clear that the dimensions of the template, i.e., the 3D model within the possibilities of the casting technology, taking into consideration additional machining, meet the requirements for accuracy. It is worth noting that none of the checked dimensions of the product or model exceeded 1% deviation from the specified dimension.

## 7. Evaluation of Cost and Time Consumption Compared to Conventional Mould Modelling Methods

According to available information from the foundry, the costs of making a model from a printout and using the classic method, i.e., the method of a wooden model, are practically the same, at least for this particular model. It should be noted that this is a relatively simple shape that does not pose a problem to create using the classic method.

However, it has to be noted that if it were a part for which the demands would be much higher in terms of shape, the printing technology would be more economically advantageous. Another advantage is the repeatability of model production.

Another plus for printed models is that it would certainly be possible to use these models for models of the sprue system as well. Here, computational models for the optimization of the inlet system could be used to the full because most of the shapes would already be physically solvable.

The last question of time requirement also comes out very favourably. Of course, everything depends on the capacity of the printer and its use. But if we were to consider the production time itself, there is certainly a big saving in time for the production of the model. The very process of transferring data (geometry) using a digital path saves time.

## 8. Conclusions

The use of this technology can significantly reduce the cost of casting production. There is also a reduction in the delivery time of the finished part. The production time of model decreased from a few days (up to one week) to a maximum of 2 days. In practice, the need to create a wooden model is eliminated. This activity was very demanding in terms of the qualification of the workers and they were only able to accept changes in the design proposal to a limited extent.

The use of 3D printing presupposes the use of 3D CAD data as input for a 3D printer. These data in the form of a digital twin of the final product (in our case the turbine inlet cone) were used as input for a 3D printer after the necessary adjustments. This printer then directly printed the model for moulding. As part of the research, an HP 4200 powder printer using PA12 GB material was used. This material was selected for its suitable mechanical properties, such as high Young modulus and long-term dimension stability. The change in dimensions with respect to time reached a maximum of one percent, which was verified by a long-term laboratory test. As part of the test, the dimensions of the 3D printed part were analysed over several months. This dimensional stability is not possible with wood because it dries out. It was verified that the 3D-printed model had a sufficient accuracy. The measured difference compared to the 3D CAD model was around 1 mm (depending on the analysed dimension). Future research will be focussed on the usage of various polymer materials as a casting model and also on cost reduction. It is obvious that the dimensional accuracy of this method is sufficient compared to commonly used techniques. There is a big challenge connected with the scaling up of this approach, focussed on the number of available 3D printing capacities. There must be an enlarged number of specialized 3D printing companies, which will be able to deliver the 3D-printed model in a short time. The environmental impact of this method must also be discussed. There is no waste from this additive technology and number of felled trees can be limited. On the other hand, the production of polymer material is highly economically demanding.

## Figures and Tables

**Table 1:** Analysed dimensions.

Dimension Description	Value in 3D Model	3D Printed Value	Value in Casting	Difference 3D Model/3D Print	Difference 3D Model/Casting
Outer diameter at cone exit	220 mm	220.9 mm	220.5 mm	+0.9 mm	+0.5 mm
Inside diameter at cone exit	192.7 mm	191.8 mm	191.5 mm	−0.9 mm	−1.2 mm
Outside diameter of cone base	456.8 mm	458.7 mm	457.3 mm	+1.9 mm	−0.5 mm
Total height of the cone	249.9 mm	250.7 mm	249.1 mm	+0.8 mm	−0.8 mm

## Data Availability

Data are contained within the article.
